# Preventing intimal thickening of vein grafts in vein artery bypass using STAT-3 siRNA

**DOI:** 10.1186/1479-5876-10-2

**Published:** 2012-01-04

**Authors:** Jiangbin Sun, Jinhua Zheng, Kaitelynne H Ling, Keyan Zhao, Zhongshang Xie, Bo Li, Tiance Wang, Zhicheng Zhu, Amit N Patel, Weiping Min, Kexiang Liu, Xiufen Zheng

**Affiliations:** 1 Department of Cardiovascular Surgery, The Second Hospital, Jilin University, Chang Chun, China; 2 The Fourth Hospital of Harbin Medical University, Harbin, China; 3 University of Western Ontario, London, Ontario, Canada; 4 Division of Surgery, University of Utah, Salt Lake City, UT 84132, USA; 5 Lawson Institute of Health Research, London, Ontario, Canada; 6 Xinjiang University, Urmuqi,China

**Keywords:** STAT-3, siRNA, vascular smooth muscle cells (VSMCs), intimal thickening

## Abstract

**Background:**

Proliferation and migration of vascular smooth muscle cells (VSMCs) play a key role in neointimal formation which leads to restenosis of vein graft in venous bypass. STAT-3 is a transcription factor associated with cell proliferation. We hypothesized that silencing of STAT-3 by siRNA will inhibit proliferation of VSMCs and attenuate intimal thickening.

**Methods:**

Rat VSMCs were isolated and cultured in vitro by applying tissue piece inoculation methods. VSMCs were transfected with STAT 3 siRNA using lipofectamine 2000. In vitro proliferation of VSMC was quantified by the MTT assay, while in vivo assessment was performed in a venous transplantation model. In vivo delivery of STAT-3 siRNA plasmid or scramble plasmid was performed by admixing with liposomes 2000 and transfected into the vein graft by bioprotein gel applied onto the adventitia. Rat jugular vein-carotid artery bypass was performed. On day 3 and7 after grafting, the vein grafts were extracted, and analyzed morphologically by haematoxylin eosin (H&E), and assessed by immunohistochemistry for expression of Ki-67 and proliferating cell nuclear antigen (PCNA). Western-blot and reverse transcriptase polymerase chain reaction (RT-PCR) were used to detect the protein and mRNA expression in vivo and in vitro. Cell apoptosis in vein grafts was detected by TUNEL assay.

**Results:**

MTT assay shows that the proliferation of VSMCs in the STAT-3 siRNA treated group was inhibited. On day 7 after operation, a reduced number of Ki-67 and PCNA positive cells were observed in the neointima of the vein graft in the STAT-3 siRNA treated group as compared to the scramble control. The PCNA index in the control group (31.3 ± 4.7) was higher than that in the STAT-3 siRNA treated group (23.3 ± 2.8) (P < 0.05) on 7d. The neointima in the experimental group(0.45 ± 0.04 μm) was thinner than that in the control group(0.86 ± 0.05 μm) (P < 0.05).Compared with the control group, the protein and mRNA levels in the experimental group in vivo and in vitro decreased significantly. Down regulation of STAT-3 with siRNA resulted in a reduced expression of Bcl-2 and cyclin D1. However, apoptotic cells were not obviously found in all grafts on day 3 and 7 post surgery.

**Conclusions:**

The STAT-3 siRNA can inhibit the proliferation of VSMCs in vivo and in vitro and attenuate neointimal formation.

## Introduction

Cardiovascular disease caused by atherosclerosis is a major cause of death world-wide. Bypass-surgery is one of most effective options to save the lives of cardiovascular patients. Veins are frequently used and have superior results compared to synthetic conduits [1]. However, recent studies have demonstrated that the primary patency of vein grafts was less than 60-80% within the first year after surgery treatment, and 50% of vein grafts develop restenosis within ten years [2-6]. A number of clinical and experimental studies have demonstrated that intimal thickening may lead to the restenosis of the vein graft [7,8]. Proliferation and migration of vascular smooth muscle cells may be a key contributor to neointima formation [9], mediated by a complex interaction of a variety of growth regulatory molecules [8,10,11]. Signal transducer and activator of transcription-3 (STAT-3) is one of these molecules involved in VSMC growth and motility induced by receptor tyrosine kinase and G protein- coupled receptor agonist [12-17]. In cultured rat VSMCs, STAT-3 activation is substantially involved in angiotensin II (AII) or platelet-derived growth factor (PDGF)-induced proliferation [18,19]. These studies suggest that STAT-3 represents a promising molecular target for inhibition of vein graft restenosis. Thus there is a strong interest in developing strategies for preventing cell proliferation and migration in order to improve the patency of venous grafts.

In order to prevent restenosis of vein grafts, extensive studies have been conducted to modulate gene expression associated with cell proliferation and growth using small molecule drugs and gene therapies by over-expression or knock-down strategies [20-25]. Among these knock-down strategies siRNA has been used to successfully mediate gene expression in vein grafts [26-30]. However, to our knowledge, the use of siRNA to inhibit STAT3 in this context has not been investigated. In this study, we demonstrated a protective effect of STAT-3 siRNA on intimal thickening in vein bypass surgery, which was associated with reduction of VSMC proliferation.

## Materials and methods

### Cell cultures

Aortic VSMCs were prepared as described previously [31]. Briefly, aortas from anesthetized rats were removed using sterile technique and placed in DMEM containing penicillin (100 U/ml) and streptomycin (100 g/ml). Vessels were cleaned of perivascular adipose tissue and cut longitudinally, and the endothelium and the adventitial tissues were scraped free using a sterile cotton swab. The tissue was incubated in DMEM for 10-15 min at room temperature. Medial smooth muscle cells were digested in DMEM containing collagenase subtype (I) (1 mg/ml, Roche, USA) for 4 hrs at 37°C. The resulting solution was centrifuged, and the pellet was resuspended in DMEM containing 20% FBS and then seeded onto 100-mm culture dishes. These cells were considered passage 0. Cells between passages 5 and 8 were used. The purity of cells (> 90%) was confirmed by examining the SMC-specific marker alpha- smooth muscle (alpha-SM) actin by flow cytometry.

### Transfection of VSMCs with siRNA

VSMCs (2 × 10^5 ^cells per well) were plated on 6-well plate one day before transfection. On the transfection day, cells were transfected with either 2 μg STAT-3 siRNA or scramble siRNA using lipofectamine 2000 with a 1:2 ratio, as described by the manufacturer's instruction. 48 h or 72 h after transfection, cells were used for subsequent experiments.

### Measurement of Cell Proliferation by Methylthiazoletetrazolium (MTT) Assay

Transfected and control VSMCs (2 × 10^5 ^cells per well) were seeded in 6-well culture plates and cultured for 72 hrs, 20 μl MTT solution (5 mg/mL) in phosphate-buffered saline (PBS) was added to the cultured cells. After 4 hrs of incubation, 100 μL of dimethyl sulfoxide (DMSO) was added to dissolve the crystals. The absorbance of the solution was read at 570 nm. Each concentration was assayed in triplicate.

### Western Blot Analysis

Soluble proteins were extracted from cultured cells using whole cell lysis buffer (5 mM Tris-HCl, 0.1 mM EDTA, 0.1% 10% (v/v) SDS, 1% IGEPAL, 10% (v/v) protease inhibitor cocktail (Sigma Chemical), pH7.5). The vein graft samples were incised and homogenized with the above whole cell lysis buffer. Following centrifugation, supernatant was collected and total protein concentration was determined by the BCA protein assay (Pierce, Rockford, IL). 20 μg protein was separated on 12% SDS-PAGE and electro-transferred to nitrocellulose membranes (Beyotime). Membranes were blocked by 5% non-fat dry milk in TBS-T (20 mM Tris, pH 7.6, and 137 mM NaCl containing 0.1% Tween-20) for 1 hr at room temperature and then incubated with primary antibodies anti-rat STAT-3 rabbit polyclonal antibody (BIOS) a, anti-rat Bcl-2 rabbit polyclonal antibody (BIOS), and anti-rat CyclinD1 rabbit polyclonal antibody, at a dilution of 1:500 (BIOS) for 2 hrs. Then the membrane was washed and incubated with secondary antibody (goat anti-rabbit, Santa Cruz) for an hour. Anti-β-actin (Sigma), as a loading control was used at a dilution of 1:3000.

### Total RNA Extraction and Reverse transcript (RT) PCR

Total RNA was isolated from VSMCs or tissue using Trizol (Invitrogen) according to the manufacturer's instruction. Isolated RNA (5 μg) was converted into cDNA with random primers and gene expression was detected using semi-quantitative PCR system (TAKARA). PCR was performed in a Perkin-Elmer GeneAMP PCR system 9700 for 30 cycles with a 58°C annealing temperature. Primer sequences for rat STAT-3, β-actin, Bcl-2 and cyclin D1 were:

STAT-3-F: 5'- TTG CCA GTT GTG GTG ATC -3', STAT-3-R: 5'- AGA CCC AGA AGG AGA AGC -3'; β-actin-F: 5' CTGGGACGACATGGAGAAAA 3', β-actin-R: 5' AAGGAAGGCTGGAAGAGTGC 3'; Bcl-2-F: 5'-GGGAGAACAGGGTACGATAA-3', Bcl-2-R: 5'-CCCACCGAACTCAAAGAA -3'; Cyclin D1-F: 5'CGC CTT CCG TTT CTT ACT TCA 3', CyclinD1R: 5'AAC TTC TCG GCA GTC AGG GGA 3'. The gene expression of STAT-3, Bcl-2 or Cyclin D1 was normalized to β-actin.

### Animal model and surgical procedure

Male wistar rats (250-350 g) were purchased from Kuming Animal Centre and were fed with standard rat chow and tap water ad libitum. All surgical procedures were ethically approved by The UWO Animal Use Subcommittee (2011-054) and The Jilin University Animal Use Subcommittee (2008-0005).

Under the microscope we interposed the left jugular vein into the left common carotid artery. Briefly, rats were anesthetized by intraperitoneal administration of 30 mg/kg of sodium pentobarbital. The left carotid artery and jugular vein were dissected via a vertical midline neck incision, and all side branches were carefully ligated with 4-0 silk suture and divided. After intravenous heparinization (1.5 mg/kg), a one-centimeter segment of jugular vein was removed and flushed with saline solution containing heparin (5 u/ml) and papaverine (0.5 mg/ml), then immersed in this solution for 5 minutes. A microvascular clip was placed at the proximal and distal end of the left common carotid artery, a part of which was cut between the two clips. The jugular vein graft was anastomosed into the carotid artery in a reverse end-to-end interposed fashion by using 11-0 polypropylene sutures. The STAT-3 siRNA or scramble siRNA with lipofectamine 2000 mixture were added into bioprotein gel and siRNA containing bioprotein gel was evenly spread onto the adventitial of the vein graft. Skin was closed with 4-0 silk suture.

### Morphometric analysis

On day 3 and 7 after the operative procedure, the rats were sacrificed to harvest the veins. The arterialized veins of rats were perfusion-fixed at 100 mm Hg via left ventricle, excised, and embedded in paraffin. 2 μm serial sections of the transverse vein graft rings were cut from each paraffin block and stained with haematoxylin and eosin (H&E) and were subjected to morphometry for assessing the intima/media area ratio (I/M ratio) and thickness of intima. The thickness of the vessel wall was determined by measuring 4 regions of a section along a cross, and recorded in micrometers (means ± SEM).

### Immunohistochemistry staining

2 μm sections were sequentially treated with 0.3% hydrogen peroxide in PBS for 30 minutes and 10% normal goat serum in 0.05 M PBS for 30 min. The sections were then incubated with diluted mouse, rabbit anti-Ki-67 (1: 100, Abcam, Cambridge, UK), and PCNA antibody (BIOS) for 30 min. Then, the sections were exposed to biotinylated goat anti-rabbit IgG (1: 200, Vector Laboratories, Burlingame, CA, USA) and streptavidin peroxidase complex (1: 200, Vector Laboratories). The sections were visualized with 3, 3'-diaminobenzidine tetrahydrochloride in 0.1 M Tris-HCl buffer and mounted on gelatin-coated slides. The number of PCNA-positive or Ki67-positive neointimal and medial cells was counted in six high-power fields from each of two sections per graft. The proliferative index was expressed as the percentage of PCNA-positive nuclei (total PCNA-positive cells/total cell count ×100%) and the percentage of Ki 67 positive nuclei (total Ki67-positive cells/total cell count ×100%), respectively.

### Terminal transferase-mediated dUTP nick end labeling (TUNEL) assay

Formalin-fixed sections were deparaffinized in 2 changes of xylene for 5 minutes each, and hydrated with 2 changes of 100% ethanol for 3 minutes each, and 95% ethanol for 1 minute. Deparaffinized sections were pretreated with proteinase K for 15 min at 37°C and washed with PBS two times. Pretreated sections were incubated with the TUNEL reaction mixture (Roche, Germany) for 1 hr at 37°C in a humidified chamber, followed by washing with PBS three times. Sections were incubated with 5% BSA for 20 min and POD for 30 min at t 37°C. Finally, sections were developed with DAB and counterstained with Hematoxylin. Apoptotic cells that appeared as brown color were visualized with a microscope.

### Statistical analyses

Data are shown as means ± SEM. Statistical analyses were performed using student t-test for comparison. A p 0.05 was considered to be statistically significant.

## Results

### 1. STAT-3 knockdown decreased the proliferation of VSMCs

**Figure 1:**
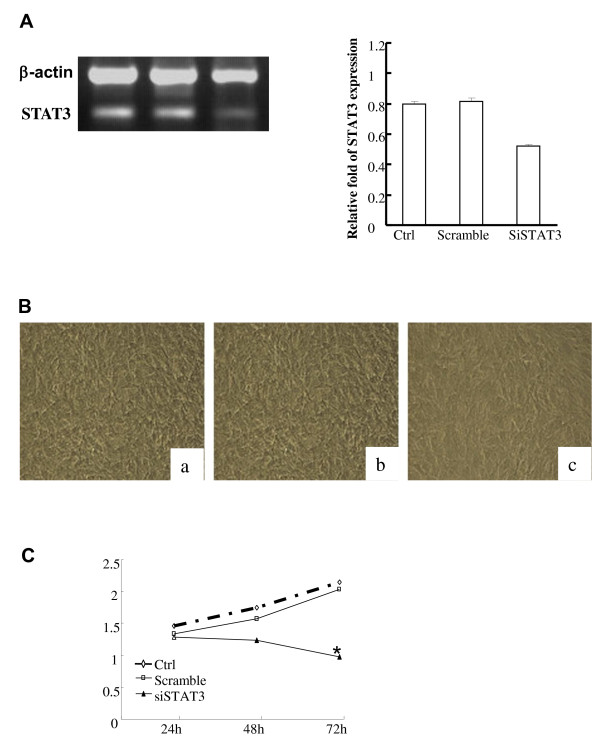
**Gene silencing of STAT-3 and its effect on cell proliferation**. *(A)*****Gene silencing of STAT-3. VSMCs were cultured and transfected with 2 μg STAT-3 siRNA (siSTAT-3) or scramble siRNA (Scramble) using lipofectamine 2000 as described in the Material & Methods. 72 hrs after transfection, cells were collected to extract RNA. STAT-3 gene expression was detected by RT-PCR. β-actin was used as an internal control. Control was cells without transfection. The intensity of band was measured. The relative target gene expression was calculated versus β-actin. *(B) *siRNA effect on the morphology feature of VMSCs. VMSCs were transfected as above.72 hrs after transfection, cells were submitted to take picture under microscope.(a) cells were not transfected; (b) cells were transfected with scramble siRNA; (c) cells were transfected with STAT-3 siRNA. *(C)*****Cell proliferation. VMSCs were transfected as above. 72 hrs after transfection, cells were used to perform MTT Assay as described in the Material & Methods. Data are representative of three independent experiments.*Different from cells treated with control siRNA or wild type (WT) VSMCs (* *p *< 0.05, student *t*-test).

In order to investigate the effect of STAT-3 siRNA on VSMCs, we first validated the gene silencing efficacy of STAT-3 siRNA. VSMCs were cultured and transfected with either STAT-3 siRNA, or scramble siRNA, using lipofectamine 2000 as described in the Methods & Materials. 72 h after transfection, cells were collected for extraction of total RNA to examine gene expression. The expression of STAT-3 gene was determined by semi-quantitative PCR. As shown in Figure 1A, 72 h after transfection the gene expression of STAT-3 in the cells transfected with STAT-3 siRNA was much lower than that in the scramble siRNA transfected group or untransfected VSMCs.

Next, we examined morphological features of VSMCs after the STAT-3 gene was silenced. Under microscope, we found that in the first 24 h after transfection, cells in all group exhibited healthy growth. 48 h after transfection, VSMCs transfected with STAT-3 siRNA started to change their morphological features, while cells in the control group still demonstrated rigorous growth. As shown in Figure 1B, 72 h after transfection, the VSMCs in both untreated group and the scramble siRNA treated group grew in a healthy and rigorous fashion, tightly attached to the bottom of the plate, in a well-formed spindle shape with clear and intermediate nucleus. In contrast, the VSMCs in the group treated with STAT-3 siRNA grew slowly and cells started to shrink with an oval shape. The density of confluence of SVMCs in the STAT-3 treated group was lower than that of control groups. Importantly, VSMCs in the STAT-3 siRNA group lost their intrinsic form and proliferated slowly with a pyknosis nucleus and some of them even floated in the culture medium. The number of cells in the STAT-3 siRNA treated group was significantly lower than that of the control and scramble plasmid group, despite the same numbers of cells being plated for transfection.

To test the effect of STAT-3 siRNA on VSMCs' proliferation, VSMCs were transfected with STAT-3 siRNA. 48 h, or 72 h after transfection, an MTT assay was performed to determine the cell proliferation of VSMCs. As shown in Figure 1C, the proliferation of VSMCs in the STAT-3 siRNA treated group was significantly inhibited in comparison to the control groups, indicating that down regulation of STAT-3 expression inhibits the growth and proliferation of VSMCs.

### 2. STAT-3 siRNA reduces the expression of STAT-3 downstream molecules Bcl-2 and cyclinD1

**Figure 2:**
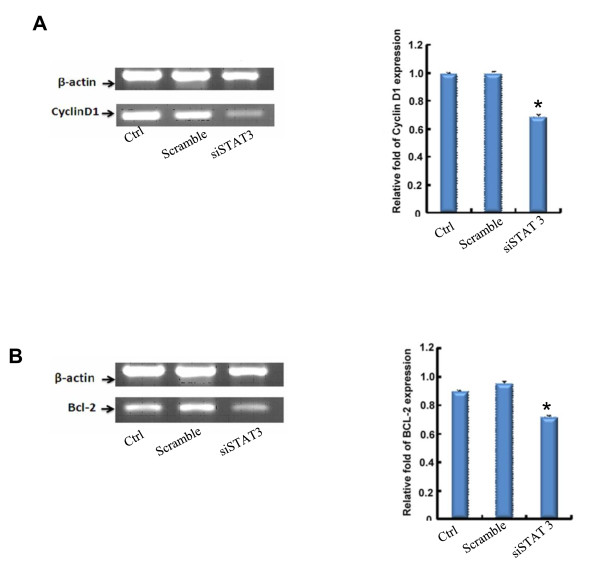
**Bcl-2 and cyclin D1 expression in STAT-3 siRNA transfected VMSCs**. VMSCs were transfected as described in Figure 1. 72 hrs after transfection, cells were collected to extract RNA. The gene expression of Bcl-2 and cyclin D1 was detected by RT-PCR and relative target gene expression was calculated as described in Figure 1. Values are means ± SEM. Data are representative of three independent experiments.*Different from cells treated with control siRNA or wild type (WT) VSMCs (*p < 0.05, student t-test).

In the STAT-3 pathway, downstream molecules of STAT-3 such as Bcl-2 and cyclin D1 regulate proliferation, differentiation and apoptosis of cells. Knocking down the gene expression of STAT-3 with STAT-3 siRNA may affect the gene expression of Bcl-2 and cyclin D1, which leads to the arrest of cell proliferation. 72 hrs after gene transfection, the expression of Bcl-2 and cyclin D1 was detected by RT-PCR at the mRNA level. As shown in Figure 2, STAT-3 siRNA significantly reduced the gene expression of Bcl-2 and cyclin D1 at the mRNA level.

### 3. STAT-3 siRNA attenuated intimal thickening

**Figure 3:**
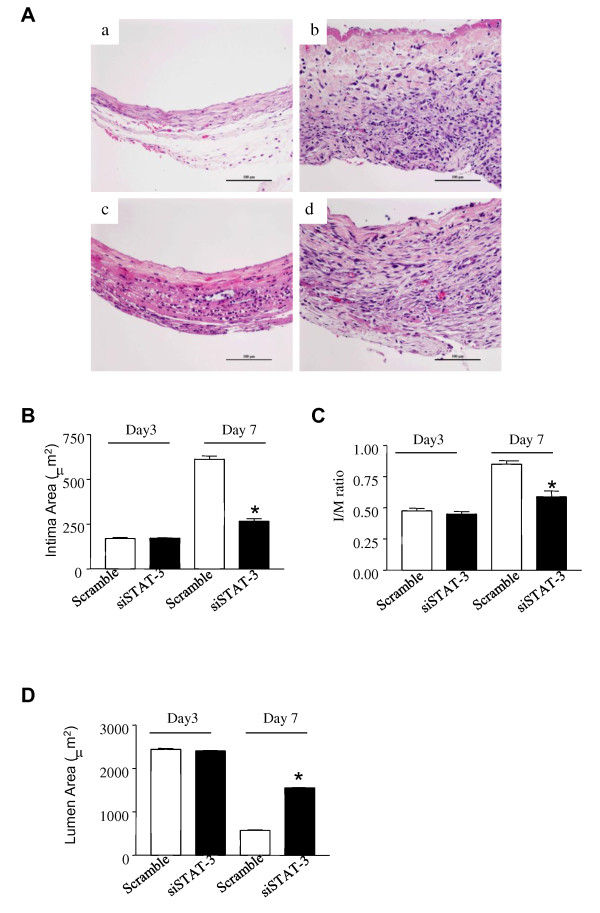
**STAT-3 siRNA reduced intimal thickening of vein graft**. (*A*) Photomicrographs of representative cross sections from vein grafts. Left jugular vein were harvested, interposed into the left common carotid artery, and then treated with either STAT-3 siRNA (siSTAT-3) or scramble siRNA (Scramble) before suture as described in the Material & Methods. The interposed veins were collected and applied to HE Staining on day 3 and 7 after operation. Bars indicate 100 μm. (a&b): Scramble siRNA treatment; (c &d): STAT-3 siRNA. (a) &(c): day 3; (b) & (d): day 7. (B)Intima area of vein grafts. (C) I/M ratios. (D)Lumen area of vein grafts. Values are mean ± SEM of five mice in each group; *P < 0.05, STAT-3 siRNA vs. scramble siRNA group.

Neointimal formation involves proliferation of vascular smooth muscle cells [9]. The in vitro studies showed that STAT-3 siRNA is capable of knocking down the expression of STAT-3, resulting in reduction in the expression of Bcl-2 and cyclin D1 and in an inhibition of cell proliferation. This positive result prompted us to test the protective effect of STAT-3 siRNA on intimal thickening in a rat vein bypass surgery model comprising of interposing the left rat jugular vein into the left rat common carotid artery. STAT-3 siRNA or control siRNA plus lipofectamine 2000 were mixed with Bioprotein gel and then evenly spread onto the adventitial of the vein graft after implantation of the jugular vein. At day 3 and 7 after the operative procedure, the interposed vein grafts containing an adjacent section of the left carotid artery were harvested and applied to H&E staining. On day 3 after the operation, there was no obvious sign indicating the intimal thickening of vessels in either the control group or STAT-3 siRNA treated group. Both groups showed inflammatory cell infiltration in the veins with thin wall (Figure 3A (a) and 3(c)). On day 7 post operation, the intimal thickening peaked in the control group, with the increased accumulation of polymorphonuclear leucocytes and mononuclear cells in the adventitia of the outer wall (Figure 3A (b)). In the STAT-3 siRNA treated group, the intima was 0.45 ± 0.05 μm thick, while 0.86 ± 0.04 μm in the control group (Table 1). STAT-3 siRNA attenuated intimal thickening and reduced the infiltration of inflammatory cells compared with control group (Figure 3A(d)). The neointimal area in the STAT-3 siRNA treated group was significantly lower compared with the control siRNA group (265.95 ± 15.25 vs. 611.58 ± 18.93 μm^2^, P < 0.05; Figure 3B) on day 7 post operation. Also, the I/M ratio was significantly decreased in the STAT-3 siRNA treated group compared with the control siRNA group (0.58 ± 0.04. vs. 0.85 ± 0.03, P < 0.05; Figure 3C). The lumen area in the STAT-s siRNA treated group was significantly higher than that in control group (574.13 ± 12.53 vs 1555.17 ± 7.24 μm^2^, P < 0.05; Figure 3D).

**Table 1:** STAT-3 siRNA Attenuated Intima Thickening of Vein Graft

Group	Intima thickness (μm) (means ± SEM)	
	
	Day3	Day 7
Scramble siRNA	o.34 ± 0.04	0.86 ± 0.04

STAT-3 siRNA	0.35 ± 0.04	0.45 ± 0.05*

*P < 0.01, the STAT-3 siRNA group versus the scramble siRNA group

### 4. Proliferation of VSMCs in intima

**Figure 4:**
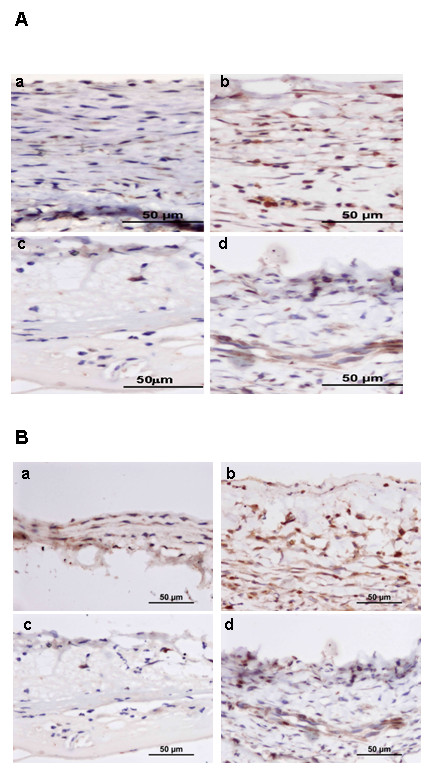
**STAT-3 siRNA prevent VMSCs proliferation in vivo**. Left jugular vein was interposed into the left common carotid artery and treated with either STAT-3 siRNA or scramble siRNA as described in the Material & Methods. The interposed veins were collected and applied to immunohistochemistry Staining with PCNA (A) and Ki-67 (B) on day 3 and 7 after operation as described in the Material & Methods. (a &b): Scramble siRNA treatment; (c &d): STAT-3 siRNA. (a) &(c): day 3; (b) & (d): day 7. Data are representative of five mice in each group.

Proliferating cell nuclear antigen (PCNA) is expressed in the late G1 (presynthetic), S (DNA synthetic), and G2 (pre-mitotic) phase of the cell cycle, serving as a marker for proliferating cells. Therefore, immunohistochemistry staining with PCNA was used to determine the proliferation of VSMCs under arterialized environment. Many PCNA-positive cells were found in the vein graft of the scramble siRNA group, while far less VSMCs existed in the STAT-3 siRNA group (Figure 4A). The PCNA index in the scramble siRNA group was significantly higher than in the STAT-3 siRNA group (Table 2). To further confirm VSMCs' proliferation, we stained vein grafts with Ki67 which is strictly associated with cell proliferation. There were fewer Ki67 positive cells found in the STAT-3 siRNA treated group as compared with the control siRNA group (Figure 4B). The Ki 67 positive cells were counted under a microscope and Ki67 index were calculated. As shown in Table 3, the Ki67 index in the scramble siRNA group was significantly higher than in the STAT-3 siRNA group.

**Table 2:** PCNA Index of Vein Grafts

Group	PCNA index of vein graft (means ± SEM)	
	
	Day3	Day7
Control	12.1 ± 2.5	31.3 ± 4.7

STAT-3 siRNA	7.3 ± 0.2	23.3 ± 2.8*

*P < 0.01, the STAT-3 siRNA group versus the scramble siRNA group

**Table 3:** Ki 67 Index of Vein Grafts

Group	Ki 67 index of vein graft (means ± SEM)	
	
	Day3	Day7
Control	6.6 ± 0.97	12.8 ± 0.95

STAT-3 siRNA	2.5 ± 0.5**	8.3 ± 1.9**

**P < 0.001, the STAT-3 siRNA group versus the scramble siRNA group

### 5. Expression of STAT-3, Bcl-2 and cyclin D1 in vein graft

**Figure 5:**
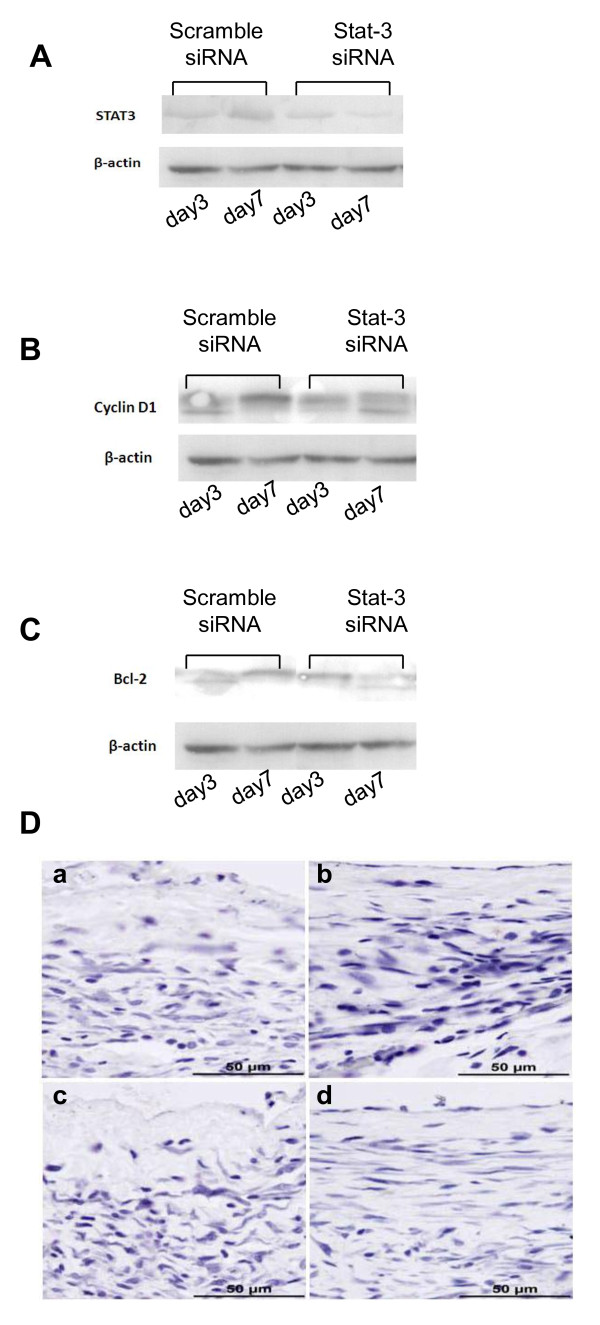
**Silencing STAT-3 with STAT-3 siRNA down regulated Bcl-2 and cyclin D1 expression**. Left jugular vein (n = 8) was treated with either STAT-3 siRNA or scramble siRNA and interposed the left jugular vein into the left common carotid artery as described in the Materials& Methods. The interposed veins were collected and total protein was extracted from above vein graft. The expression of Bcl-2 and cyclin D1 was detected by Western Blot as described in the Material & Methods. (A) Gene expression of STAT-3; (B) Gene expression of cyclin D1; (C) Gene expression of Bcl-2; (D) TUNEL assay. Vein grafts were sectioned and subjected to detect apoptotic cells by TUNEL assay as described in the Material & Methods. (a & b): Scramble siRNA treatment; (c & d): STAT-3 siRNA. (a) &(c): day 3; (b) & (d): day 7.

To elucidate whether STAT-3 gene silencing by STAT-3 siRNA was associated with suppression of Bcl-2 and cyclin-D in vivo, expression of these proteins in the vein graft was assessed by Western Blot. Protein samples were extracted from the homogenates of the vein grafts (n = 8) harvested on day 3 and 7 post operation. As shown in Figure 5A, B and 5C, STAT-3, Bcl-2 and cyclin D1 were expressed in all samples harvested at different time points. On day 3 after operation, STAT-3, Bcl-2 and cyclin D1 were expressed in the tissues at a very low level. On day 7 the gene expression of STAT-3, Bcl-2 and cyclin D1 increased two-fold in the control group, while gene expression of STAT-3, Bcl-2 and cyclin D1 in the STAT-3 siRNA treated group did not significantly increase. In addition, we examined cell apoptosis in the vein grafts using TUNEL assay. There were no apoptotic cells detected in both STAT-3 siRNA treated group and control groups at day 3 and 7 post surgery (Figure 5D).

## Discussion

In this study, we demonstrated that STAT-3 siRNA not only decreased the proliferation of VSMCs in vitro (Figure 1C) and in vivo (Figure 4), but also subsequently prevented neointima formation in an experimental vein graft interposed model (Figure 3 and Table 1). Down regulation of STAT-3 with siRNA reduced the expression of the anti-apoptotic Bcl family protein Bcl-2 and pro-proliferation protein cyclin D1 (Figure 2 and 5) leading to a decrease of VSMCs' proliferation (Figure 1C and Figure 4, Table 2 and Table 3). To our knowledge, this is the first demonstration that STAT-3 siRNA is capable of reducing intimal thickening of experimental vein grafts.

In venous bypass surgery, the formation of neointima is an important etiologic factor in vein graft restenosis. Numerous studies have demonstrated the molecular mechanisms underlying restenosis [9,24,32]. It has been well established that accumulation and proliferation of VSMCs either migrating from the deeper medial layer of vessels or from the circulating hematopoietic stem cells is one of key events in the formation of neointima [8,33]. The proliferation of VSMCs in response to injury is regulated by different stimuli, including growth factors, inflammatory cytokines and other proteins which are associated with cell proliferation, such as STAT family and cyclin-dependent kinases expressed in VSMCs [11,15,17,34]. To date, a number of strategies have been investigated for prevention of VSMCs accumulation and migration. Gene therapy served as a major therapy targeting different signaling pathways involved in the VSMCs proliferation and migration for vascular grafts stenosis [25,35-38]. Stephan et al infected porcine VSMCs with beta-interferon using adenoviral means, resulting in reduced cell proliferation in vitro and in vivo [39]. Over-expression of wild type of p53 in the human saphenous vein infected by an adenovirus encoding p53 gene induced apoptosis of VSMCs and inhibited VSMCs migration, resulting in a reduction in intimal thickening [11,40]. In this study, we silenced the STAT-3 gene by RNA interference, which is considered one of the most powerful methods of inhibiting gene expression. We transfected VSMCs with STAT-3 siRNA and silenced the STAT-3 gene. The down regulation of the STAT-3 gene reduced the expression of its downstream molecules Bcl-2 and cyclin D1 leading to inhibition of VSMCs proliferation and induction of apoptosis. In a rat venous bypass model, we found that the intima thickness reached its peak on day 7 post operative procedure, and begin to decline from week two. The expression of STAT-3 in vein grafts was up-regulated on day 7 after surgery. Treating the vein graft by coating the adventitia with STAT-3 siRNA/lipofectamine and bioprotein gel decreased STAT-3 expression. The intima of vein grafts treated with STAT-3 siRNA did not lead to apparent thickening, only with scabrosity, while the intima of vein grafts in the control group thickened up to three times on day 3. On day 7, the ratio of intima area/media area was 0.85 ± 0.03 in the scramble group and 0.58 ± 0.04 in the STAT-3 siRNA group respectively.

PCNA and Ki-67 were used to measure the proliferative ability of cells in paraffin embedded tissue [41]. Zwolak demonstrated that, in vein graft models, the SMC proliferation rate is at its highest within the intima from day seven up to three weeks, followed by a later decline [42,43]. Our results demonstrated that the PCNA and Ki-67 positive cells was significantly less in vein grafts of the STAT-3 siRNA treatment group on day 7 compared with those of the scramble group (Figure 4). It is conceivable that migration and proliferation of vascular smooth muscle cells were inhibited by STAT-3 siRNA.

Accumulating evidence suggests that STATs can be activated by various cytokines and growth factors, and that activated STAT-3 forms homodimer and heterodimer cis-inducible factor complexes which then translocate into the nucleus and activate transcription of early growth response genes, including Jun B, IRF-1, Cyclin D1, and anti-apoptotic factors Bcl-xL and Bcl-2, leading to proliferation, differentiation, and apoptosis regulation [44,45]. Marrero reported that, in cultured rat VSMCs, JAK2-mediated STAT-3 activation is substantially involved in the angiotensin II (AII) - or platelet-derived growth factor (PDGF)-induced proliferation [18]. Generally, the activation of STAT-3 can only maintain for hours, but in the condition of vascular injury or neointima thickening, it can be continuously activated, especially STAT-1 and STAT-3. In balloon injury models, STAT-3 expression is induced in medial and neointimal VSMCs at days 2 and 5, respectively, and peaks at day 7 when neointimal VSMC proliferation is at a maximum, then slowed at the thickening by day 14 (data no shown). Our western-blot results showed that the protein levels of STAT-3, Bcl-2 and cyclin D1 were remarkably upregulated on day 7, while the protein levels of STAT-3 siRNA group were significantly lower than those in the scramble group.

In addition to bypass-surgery, coronary stent implantation has now become a common practice in interventional cardiology. However, restenosis also remains by far the main complication of this technique. A recent study from the Wang group has shown that treatment with siRNA targeting CREB binding protein markedly reduced CBP expression and significantly decreased neointimal formation [46]. The Letourneur's group reported that implantation of stents coated with siRNA targeting MMP-2 which is associated with restenosis can induce an uptake of siRNA into the arterial wall and decrease pro-MMP2 activity [47], suggesting that stents coated with siRNA represent as a therapeutic approach for cardiovascular diseases.

## Conclusions

Our findings demonstrate that STAT-3 siRNA has a potent effect on VSMC proliferation by regulating expression of STAT-3 and its downstream genes Bcl-2 and cyclin D1 at both mRNA and protein levels. STAT-3 is a promising target to prevent restenosis. RNA interference provides a new route to improve the patency rate for venous bypass grafts.

## Abbreviations

siRNA: small interfering RNA; VSMCs: vascular smooth muscle cells; STAT-3: Signal transducer and activator of transcription-3; PCNA: Proliferating cell nuclear antigen; AII: angiotensin II; PDG: platelet-derived growth factor.

## Competing interests

The authors declare that they have no competing interests.

## Authors' contributions

JS carried out surgery and in vivo experiments. JZ participated in vitro assay. KL participated in manuscript preparation. KX participated in gene expression. ZX participated in histopathology assessment. BL carried out in vitro assay. TW, participated in the project design and in vivo experiments. ZZ participated in the project design and in vivo experiments. AP participated in manuscript. WM helped to draft the manuscript. KL participated in the project design, coordination the experiments, and manuscript preparation. XZ participated in the project design, coordination the experiments, and manuscript preparation. All authors read and approved the final manuscript.
